# Mitochondrial Haplogroups H and J: Risk and Protective Factors for Ischemic Cardiomyopathy

**DOI:** 10.1371/journal.pone.0044128

**Published:** 2012-08-28

**Authors:** Mariana Fernández-Caggiano, Javier Barallobre-Barreiro, Ignacio Rego-Pérez, María G. Crespo-Leiro, María Jesus Paniagua, Zulaika Grillé, Francisco J. Blanco, Nieves Doménech

**Affiliations:** 1 Cardiac Biomarkers Group, Research Unit, INIBIC-Complejo Hospitalario Universitario A Coruña, A Coruña, Spain; 2 Genomic Lab, Rheumatology Division, INIBIC-Complejo Hospitalario Universitario A Coruña, A Coruña, Spain; 3 Advanced Heart Failure and Heart Transplant Unit, Cardiology Department, Complejo Hospitalario Universitario A Coruña, A Coruña, Spain; 4 Spanish Cardiovascular Research Network (RECAVA), Instituto de Salud Carlos III, Madrid, Spain; IInstituto de Investigación Hospital 12 de Octubre, Spain

## Abstract

**Background:**

Since mitochondria are the principal source of reactive oxygen species (ROS), these organelles may play an important role in ischemic cardiomyopathy (IC) development. The mitochondrial genome may influence this disease. The aim of the present study was to test the relationship between IC development and the impact of single nucleotide polymorphisms (SNPs) in mitochondrial DNA (mtDNA) defining the mitochondrial haplogroups in a population study.

**Methodology and principal findings:**

Ten major European haplogroups were identified by using the single base extension technique and by polymerase chain reaction-restriction fragment length polymorphism. Frequencies and Odds Ratios for the association between IC patients (n = 358) and healthy controls (n = 423) were calculated. No convincing associations between classical risk factors for ischemic cardiomyopathy development and haplogroups were found. However, compared to healthy controls, the prevalence of haplogroup H was significantly higher in IC patients (40.0% vs 50.0%, p-value  = 0.039) while the frequency of haplogroup J was significantly lower (11.1% vs 5.6%, p-value  = 0.048). The analysis of the SNPs characterizing the European mtDNA haplogroups showed that the m.7028C allele (40.0% vs 50.0%, p-value  = 0.005) and m.14766C allele (43.0% vs 54.2%, p-value  = 0.002) were overrepresented in IC patients, meanwhile the m.10398G allele (19.8% vs 13.1%, p-value  = 0.015) and m.4216C allele (22.2% vs 16.5%, p-value  = 0.044) were found as protective factors against IC.

**Conclusions and significance:**

Our results showed that the haplogroups H and J were found as a risk and protective factors for ischemic cardiomyopathy development, respectively.

## Introduction

Ischemic heart failure continues to be a major health problem in the Western countries [1]. An in-depth understanding of its basic pathophysiological mechanisms is necessary to provide early prognosis and better therapies for preventing and prevention of the disease. It is generally known that reactive oxygen species (ROS) are involved in various cardiovascular diseases, such as ischemic injury, coronary heart disease and congestive heart failure. An imbalance between endogenous oxidants and antioxidants results in oxidative stress, which contributes to vascular dysfunction and atherogenesis [2]. Atherosclerosis in coronary arteries is the primary cause of ischemic cardiomyopathy (IC). Inflammation and ROS production have recently been established as key mechanisms in the pathogenesis of atherosclerosis and in coronary artery disease progression [3], [4]. Because mitochondria are the principal source of ROS in cardiomyocytes, these organelles may play an important role in ischemic cardiomyopathy development.

Human mitochondrial DNA (mtDNA) encodes 37 genes. Yet, only 13 of these genes are transcripted into 13 polypeptides. They constitute essential subunits of the mitochondrial oxidative phosphorylation enzymatic complexes that generate the main source of ATP [5], [6]. The mtDNA mutations accumulated throughout human history have divided human populations into a small number of mitochondrial haplogroups. Point mutations observed in over 1% in the population are known as SNP (*Single Nucleotide Polymorphism)*. A mitochondrial haplogroup is defined as a collection of haplotypes characterized by specific SNPs [7]. Haplogroups exhibit specific polymorphisms in indigenous populations and this phenomenon has been attributed to genetic drift and/or possible climate selection [8], [9].

Although polymorphic variants are not pathogenic, they are not silent either. For example, specific mitochondrial polymorphisms are involved in the assembly of components of the mitochondrial respiratory chain [10]. Moreover, several studies have shown mitochondrial haplogroups to be associated with differences in the amount of superoxide and other ROS produced by the electron transport chain [11]. Consequently, this leads to varying amounts of oxidative stress in cells depending upon their haplogroup and therefore to differences in morbidity, mortality and longevity amongst individuals with distinct haplogroups [12], [13]. One study has reported that individuals with haplogroup J present lower oxygen consumption than other haplogroup variants [11]. This could be due to decreased efficiency of their electronic respiratory chain and therefore, a decreased in ATP and ROS production [9]. Thus, specific haplogroups may constitute either a risk or a protective factor in the origin of complex diseases and age-related diseases such as Parkinsonn`s [14], Alzheimer`s disease [15], [16], osteoarthritis [17], [18] and several cancers [19], [20]. Mitochondrial haplogroups have also been associated with increased risk of developing various cardiovascular diseases. For example, haplogroup T has been found more frequent in patients with hypertrophic cardiomyopathy [21]. Whilst haplogroup H1 [22], K [23], the Asian haplogroup N9b [24] have been reported as protective factors against ischemic stroke, the Asian haplogroups A and M7a confer a genetic risk for developing coronary atherosclerosis [25]. Conversely, it is worth noting that a large population study did not find any association between mtDNA haplogroups and ischemic heart disease in a European population [26].

The differences in mitochondrial energetic production among haplogroups could potentially affect the development of IC. This study was designed to investigate the possible association of mitochondrial haplogroups in a population of 358 IC patients and 423 controls from the Spanish population. To accomplish this, we genotyped both groups of individuals for the most common European haplogroups and examined the differences in their frequency between controls and IC patients.

## Methods

### Ethics Statement

The study was conducted according to the Spanish Law for Biomedical Research (Law 14/2007-3 of July) and complied with the Declaration of Helsinki. The study and the use of archive samples for this project were approved by the Research Ethics Committee of Galicia. The National DNA Bank, which provided DNA samples, received the approval from their own ethical committee. Written informed consent was obtained from all patients. All the samples were collected anonymously.

### Patients and Controls

This case-control followed STREGA guidelines [27]. DNA samples from 781 unrelated Spanish individuals (423 healthy controls and 358 IC patients) were analysed in this study. The ischemic cardiopathy group included 225 patients obtained from A Coruña University Hospital Cardiology Unit and 133 provided by the National DNA Bank (University of Salamanca, Spain). The control group was an age and sex matched population of donors from A Coruña University Hospital Blood Bank. Individuals in this group represented both genders and had no history of IC.

Ischemic cardiopathy was defined according to the American College of Cardiology and American Heart Association clinical standards [28]. Information about known ischemic cardiopathy risks was collected. Hypercholesterolemia was considered a risk if total cholesterol levels ≥220 mg/dl. Hypertension was defined as systolic blood pressure ≥140 mm Hg, diastolic blood pressure ≥90 mm Hg or by the use of antihypertensive medication. Diabetes mellitus was defined as a self-reported disease, use of antidiabetic drugs, or a nonfasting plasma glucose ≥11 mmol/l. Smokers were defined as current smokers.

### Assignment of mtDNA Haplogroups

Haplogroup analysis was performed taking into account a previously described protocol [18]. This strategy was based on the use of single base extension (SBE) for the assessment of European mtDNA haplogroups. The SBE assay allows identifying six SNPs that determine the most frequent European haplogroups (H, T, K, U, J, V), while the less common haplogroups (W, I, X and HV) were identified afterwards by polymerase chain reaction-restriction fragment length polymorphisms (PCR-RFLPs).

Total DNA was extracted from blood samples using QIAmp DNA Blood Mini Kit (Qiagen, Germany). Fragments containing the six target SNPs were amplified using twelve primers listed in Table 1. The multiplex PCR mixture was diluted in Reaction Buffer (Bioline, UK) on a final concentration of 0.2 mM of each deoxynucleotide (dNTP) (Bioline. UK), 1.5 mM MgCl_2_ (Bioline, UK), 0.025 U/µl of Bio Taq DNA polymerase (Bioline, UK) and 0.3 µM of each primer in a total volume of 25 µl. Isolated DNA (100 ng) was added to the mixture and amplified at 94°C for 5 minutes, 35 cycles at 95°C for 60 seconds, 55°C for 60 seconds and 72°C for 60 seconds, with a final extension at 70°C for 10 minutes. 1.5 µl of PCR products were treated with 0.6 µl ExoSap-It (Amersham, UK) followed by an activation of the enzyme at 37°C for 15 minutes and a further deactivation by incubation at 80°C for 15 minutes. Samples were stored at 4°C.

**Table 1:** Primer sequences used for in multiplex PCR, SBE and PCR-RFLP.

	Polimorphicsite	PCR primer	Position	SNPanalyzed	Restrictionenzime
**Multiplex PCR**	7028	5′-CTGACTGGCATTGTATTAGCA-3′ 5′-GTATACGGGTTCTTCGAATG-3′	6960F 7433R		
					
	14766	5′-GAGAAGGCTTAGAAGAAAACCCCAC-3′ 5′-GTGGGCGATTGATGAAAAGGC-3′	14601F 14950R		
					
	10398	5′-GGCCTATGAGTGAACTACAAAA-3′ 5′-TATTCCTAGAAGTGAGATGGT-3′	10364F 10526R		
					
	4580	5′-CCTACCACTCACCCTAGCATTAC-3′ 5′-TAGGAATGCGGTAGTAGTTAG-3′	4185F 5120R		
					
	12308	5′-CAACCCCGACATCATTACCGGGT-3′ 5′-GGGTTAACGAGGGTGGTAAGG-3′	12106F 12413R		
					
	4216	5′-CCTACCACTCACCCTAGCATTAC-3′ 5′-GCGAGCTTAGCGCTGTGATGAG-3′	4185F 4542R		
					
**Single** **Base** **Extensi** **on (SBE)**	7028	5′-ACACGACACGTAACTACGTTGTAGC-3′	7004F	m.7028C>T	
	14766	5′cgatcATGAGTGGTTAATTAATTTTATTAGGGTTA-3′	14798R	m.14766C>T	
	10398	5′-ataTATGAGTGACTACAAAAAGGATTAGA CTGA-3′	10368F	m.10398A>G	
	4580	5′-(at)7TTTTTTACCTGAGTAGGCCTAGAAA TAAACAT-3′	4548F	m.4580G>A	
	12308	5′-(tacg)5aCCATTGGTCTTAGGCCCCAA-3′	12288F	m.12308A>G	
	4216	5′-cgCCACTCACCCTAGCATTACTTATATG A-3′	4189F	m.4216T>C	
**PCR-** **RFLP**	10032	5′-CTTTGGCTTCGAAGCCGCCGCC-3′ 5′- TATTCCTAGAAGTGAGATGGT-3′	9902F 10526R	m.10034T>C	(−)AluI
					
	14465	5′-ATGCCTCAGGATACTCCTCAATAGCCAT C- 3′ 5′- CCGTGCGAGAATAATGATGTATGC-3′	14430F 14686R	m.1470T>C	(+)AccI
					
	8994	5′- TAGCCCACTTCTTACCACAAGGC-3′ 5′-GTGTGAAAACGTAGGCTTG-3′	8900F 9172R	m.8994G>A	(−)HaeIII
					

R: primer in reverse orientation; F:primer in forward orientation.

* Lower case letters indicate the unspecific nucleotides in 5′-end of the SBE primer. PCR products for RFLP analysis were digested with the corresponding restriction enzyme and digested PCR products appeared like three fragments in agarose gel.

The SBE assay consisted on annealing of 6 single primers to 6 fragments amplified with Multiplex PCR (Table 1). The 3′ end of each primer was one base shorter of the SNP site of interest. Only dideoxynucleotides (ddNTPs) were employed in the reaction. When the complementary base was incorporated by the Taq DNA polymerase, the elongation stops and the SNP site were marked. Multiplex SBE reactions were performed by adding 1.5 µl of SNaPshot® Multiplex kit (Applied biosystems, EEUU), 2.1 µl of purified PCR product and of 0.2 µM of SBE primers mixture (Table 1). Reaction volumes were adjusted to 10 µl using ddH_2_O. Thermal cycling for SBE was 96°C for 60 seconds and 25 cycles at 96°C for 20 seconds, 60°C for 5 seconds and 60°C for 30 seconds. To dispose ddNTPs, the SBE reaction products (10 µl) were treated with 1 µl of Shrimp Alkaline Phosphatase (SAP) and 2 µl of SAP Reaction Buffer (Amersham, UK). Reaction volumes were adjusted to 20 µl using ddH_2_O. The mixture was incubated at 37°C for 1 hour and SAP was inactivated in a final incubation at 75°C for 15 minutes. Samples were stored at 4°C.

Finally, 9 µl of Hi-Di™ Formamide (Applied Biosystems, EEUU), 0.5 µl of internal size standard (120 Liz Size; Aplied Biosystems, USA) and 0.5 µl of purified SBE product were mixed and denatured at 95°C for 5 minutes before loading into and ABI 3130XL genetic analyzer. The system configuration is based on a 36 cm length capillary tube filled with Performance Optimized Polymer 4 (Applied Biosystems, USA) containing urea. The default SBE run module was 22 seconds of injection time, 16 minutes of running time and a running voltage of 15KVolts. Once the runs finished, the data were analyzed using GeneMapper v3.5 software (Aplied Byosistems, USA). This software assigns the different SNPs in each locus prior to the designing of a reference sequence encompassing all the allelic variants for each locus.

The PCR-RFLP assay was performed on those samples having no haplogroup assigned after the SBE assay. The samples were amplified with the corresponding primers (Table 1) and digested depending on the nucleotide localised at polymorphic site 10398 (m.10398A>G). Samples with the m.10398G allele were tested for 10032*AluI* (10032 *AluI* positive samples were assigned to haplogroup I (m.10034C allele), and 10032 *AluI* negative samples were assigned to “others”). Samples having m.10398A allele were tested for 14465*AccI* and 8994*HaeIII*. 14465*AccI* positive samples were assigned to haplogroup X (m.14470C allele). 14465*Acc*I negative (m.14470T allele) and 8994*HaeIII* negative (m.8994A allele) samples were assigned to haplogroup W. 10–15 µl of PCR product were digested with 2 µl of the appropriate restriction enzyme (Table 1), 4 µl of buffer and ddH_2_O to reach a final volume of 40 µl. Digestion was performed at 37°C for 90 minutes. Then, the samples were stored at 4°C. To confirm that the enzyme indeed cut the amplified fragment, the digestion products were run in agarose gels and visualized using UV after SYBR-safe treatment.

### Statistical Methods

Data were analyzed using SPSS 17.0 software (IBM, USA). Haplogroup and allele frequencies in patients and controls were compared using the chi-square test from contingency tables. The Odds Ratio (OR) and 95% confidence intervals (CI) were calculated for each haplogroup. For the haplogroup analysis, each haplogroup was compared against all the other haplogroups pooled into a single group. The less frequent haplogroups I, W and X, which account for less than 10 controls/patients, were re-grouped based on common-ancestor criteria. The haplogroup HV was re-grouped as “others”. Binary logistic regression adjustment was used to test the influence of hypercholesterolemia, hypertension, diabetes mellitus and the smoking habit. Differences were considered significant when p<0.05 (2-tailed test). The Bonferroni correction for multiple comparisons was applied. Thus, p-values were multiplied by the number of outcomes (k = 8 for mtDNA haplogroups and k = 4 for mtDNA clusters) tested.

Haplogroups prevalence varies extremely, with frequencies between 40% to <5% in the general population. Assuming a haplogroup prevalence of 40% in the control group, the sample size of this study provides 80% power to detect a significant associated odds ratio ≥1.50, at 2-sides significant level of 0.05. For a haplogroup with prevalence around 20%, the sample size allows to detect significant odds ratios ≥1.64 (power  = 80%, α level of 0.05). For more rare haplogroups (prevalence around 5%), only odds ratios ≥ 2.27 will be detected as statistically significant (power  = 80%, α level of 0.05).

## Results

### No Association was Found between Mitochondrial Haplogroups and the Major Risk Factors for IC

Of the 781 Spanish subjects included in this study, 358 had been previously diagnosed with IC (case group) and 423 were controls with no history of IC. Because IC is a very common late-onset disease we chose subjects of similar ages in both groups (66.3±11.8 years for the IC group and 66.5±11.9 years for the control group). The lower proportion of women developing IC was also considered. For this, the control group (17.5% of women) was selected to have frequency similar to the case group (18.2% of women). Risk factors for ischemic cardiovascular events stratified by mitochondrial haplogroups are listed in Table S1. Remarkably, the distribution of haplogroups for the major IC risk factors was no different between controls and patients.

### Haplogroups H and J were Respectively, Risk and Protective Factors for IC

Study subjects were genotyped for the most common European descent mitochondrial haplogroups; the resulting frequencies are shown in Table 2. The frequencies obtained ranged from 50.0% for the most common haplogroup H, to 0.8% for the less prevalent haplogroup I. The haplogroup frequencies for our controls did not differ substantially from those reported in previous studies on different European populations (Table S2) [29]–[31]. Using the rapid and effective multiplex SBE assay, 89.88% of the samples were assigned to the most common European mtDNA haplogroups (H, U, J, K, T, V). The less frequent haplogroups (X, I, W) were determined for 6.02% of the samples using the classical PCR-RFLP assay. Only 4.10% of the samples remained undetermined after the analysis and they were classified as “others”.

**Table 2:** Frequencies and OR of mitochondrial haplogroups and clusters in controls and patients with IC.

		N° of individuals (%)		Total	OR [95%CI]	p-value
		Control(n = 423)	IC patients(n = 358)			
**Haplogroups**	H	169 (40.0)	179 (50.0)	348 (44.6)	1.50 [1.13–2.00]	**0.039** *
	U	73 (17.3)	58 (16.2)	131 (16.8)	0.92 [0.64–1.35]	0.694
	J	47 (11.1)	20 (5.6)	67 (8.6)	0.47 [0.28–0.82]	**0.048** *
	T	47 (11.1)	39 (10.9)	86 (11.0)	0.98 [0.62–1.53]	0.923
	K	28 (6.6)	19 (5.3)	47 (6.0)	0.79 [0.43–1.44]	0.442
	V	13 (3.1)	10 (2.8)	23 (2.9)	0.82 [0.39–2.09]	0.818
	I WX	27 (6.4)	20 (5.6)	47 (6.0)	1.13 [0.60–2.12]	0.698
	OTHERS	19 (4.5)	13 (3.6)	32 (4.1)	0.80 [0.39–1.65]	0.551
**Clusters**	HV	188 (44.4)	194 (54.2)	382 (48.9)	1.47 [1.11–1.96]	**0.027** *
	JT	94 (22.2)	59 (16.5)	153 (19.6)	0.69 [0.48–0.99]	0.044
	KU	101 (23.9)	77 (21.5)	178 (22.8)	0.88 [0.63–1.23]	0.444
	IWX	27 (6.4)	20 (5.6)	41 (6.0)	1.13 [0.60–2.12]	0.698

OR. Odds Ratio. 95%CI. Confidence Intervals. IC. Ischemic cardiomyopathy.

* Bonferroni corrected p-value <0.05/8 for haplogroups and p-value <0.05/4 for clusters. Significant differences (p-value<0.05) are indicated in bold.

The frequencies of two mitochondrial haplogroups in IC patients differed significantly from those in healthy controls. The haplogroup H was significantly overrepresented in IC patients (OR = 1.50 CI = [1.13–2.00], p<0.05) when each haplogroup was compared against the remaining pooled haplogroups. (Table 2). On the other hand, the frequency of haplogroup J in IC patients was significantly lower than in controls (OR = 0.47 CI = [0.28–0.82], p<0.05). These results suggest that haplogroup H constitutes a risk factor while haplogroup J is a protective factor for IC.

Hypercholesterolemia, hypertension, diabetes and smoking habit were significantly independently associated with IC after the multivariate logistic regression analysis (Table 3). Our results supported previous studies showing that IC development was associated with hypercholesterolemia (OR = 1.58 CI = [1.13–2.20], p<0.05), hypertension (OR = 2.01 CI = [1.46–2.76], p<0.001), diabetes (OR = 1.58 CI = [1.09–2.28], p<0.05) and cigarette smoking (OR = 2.37 CI = [1.63–3.45], p<0.001). The haplogroup H continued to be a risk factor compared with haplogroup J (OR = 0.28 CI = [0.15–0.52], p<0.001) and others (OR = 0.59 CI = [0.37–0.95] p<0.05) (Table 3). These results support the conclusion that patients with IC are overrepresented in haplogroup H compared with the haplogroup J and others.

**Table 3:** Multivariate analysis of the study groups.

	B	SEM	OR [95% CI]	p-value
Hypercholesterolemia	0.458	0.170	1.58 [1.13–2.20]	**0.023**
Hypertension	0.696	0.163	2.01 [1.46–2.76]	**0.000**
Diabetes	0.456	0.187	1.58 [1.09–2.28]	**0.011**
Smoking habit	0.864	0.191	2.37 [1.63–3.45]	**0.000**
Haplogroup H	–	–	1	–
Haplogroup U	−0.305	0.214	0.74 [0.48–1.12]	0.155
Haplogroup J	−1.259	0.310	0.28 [0.15–0.52]	**0.000**
Haplogroup T	−0.298	0.253	0.74 [0.45–1.22]	0.239
Haplogroup K	−0.292	0.330	0.75 [0.39–1.42]	0.376
OTHERS	−0.519	0.241	0.59 [0.37–0.95]	**0.031**

B. Regression coefficient. SEM. Standart error of the mean. OR. Odds Ratio. 95%CI. Confidence Intervals.

Significant differences (p-value<0.05) are indicated in bold.

Because the analyzed haplogroups share a common ancestor and several SNPs have been conserved during evolution, in our study we also examined the frequencies of clusters HV, JT, KU and IWX. Although the frequency of haplogroup J was found to be significantly different from controls, no difference was found in Cluster JT between controls and patients with IC (Table 2). However, cluster HV was found to be a risk factor for IC (OR = 1.47 CI = [1.11–1.96], p<0.05).

The analysis of the SNPs characteristic of the European mtDNA haplogroups showed that frequency of the C allele at positions 7028 and 14766 was significantly higher in patients with IC (OR = 1.50 CI = [1.13–2.00], p<0.05 and OR = 1.57 CI = [1.18–2.08], p<0.05) respectively). The nucleotide change in 7028 *locus* causes a synonymous amino acid change whilst 14766 *locus* produces a non-synonymous amino acid change in cytochrome b (p.Thr7Ile). On the other hand, the m. 10398G allele and the m.4216C allele were found as protective factors against IC (OR = 0.61 CI = [0.41–0.91], p<0.05 and OR = 0.69 CI = [0.48–0.99], p<0.05, respectively). The SNP m.10398A>G causes a non-synonymous amino acid change in NADH dehydrogenase subunit 3 (p.Thr114Ile) which is observed in haplogroups K, J and I. The SNP m.4216T>C is specific for the cluster JT and produces a non-synonymous amino acid change in NADH dehydrogenase subunit 1 (p.Tyr304His).

## Discussion

We found significant associations of mitochondrial haplogroups H and J and the incidence of ischemic cardiomyopathy in a Spanish population. Our results showed that the haplogroups H and J are respectively risk and protective factors for ischemic cardiomyopathy development.

In our study population, age and gender proportions were similar in the case and control groups. This permitted us to exclude any bias due to age or gender differences. Also, we checked the frequencies for major risk factors for ischemic cardiomyopathy (hypercholesterolemia, hypertension, diabetes and cigarette smoking) stratified by mitochondrial haplogroups showing that they were not altered. According to other studies classical risk factors for IC development were independently associated with IC [32], [33].

Several studies have demonstrated association between haplogroups and complex or aged-related diseases, including cardiovascular diseases [14]–[24]. In agreement with our findings, a recent study showed that mitochondrial haplogroup H is a risk factor for the early onset myocardial infarction [34]. Besides, Gallardo et al. described haplogroup H as a risk factor for the progress to end-stage heart failure in a Spanish population [35]. In agreement with our study they observed that the frequency of haplogroup H in 174 IC patients was 53% when they divided the allograft recipients according to aetiology. Our data support in a statistical way that haplogroup H is a risk factor for ischaemic cardiomyopathy.

Although many SNP characterised haplogroup H, the m.14766C allele constituted a risk factor for the development of ischemia in our study. The SNP m.14766C>T causes the amino acid substitution of a threonine for an isoleucine at site 7 in cytochrome b, thus possibly affecting protein complex II and the electron transport chain. A computational approach showed that the region around cytochrome site 7 is more open, less globular and less compact due to the presence of a threonine. Consequently, the efficiency of the ETC is expected to be higher in haplogroup H cells [36]. The allele m.7028C, which also characterizes the haplogroup H, was also found overrepresented in our study. However, the SNP m.7028C>T causes a synonymous amino acid change, therefore this SNP is not responsible of phenotypic effect that define haplogroup H as a risk factor for IC development. On the other hand, Rosa et al. reported the sub-haplogroup H1 as a protective factor for ischemic stroke [22]. Because sub-haplogroup H1 is defined at the 7028 mtDNA position by the same SNP as haplogroup H, other polymorphisms present in sub-haplogroup H1 might produce different phenotypes. A case-controlled study including 920 patients and 522 controls reported haplogroup N9b specific polymorphisms m.11016G>A (p.Ser86Asn in NADH dehydrogenase 4) and m.13183A>G (p.Ile283Val in NADH dehydrogenase 5) confer resistance against ischemic events [24]. The haplogroup J specific polymorphisms that we report here to be protective factors are not present in haplogroup N9b. On the contrary, haplogroup J is characterized by several missense mutations affecting genes encoding cytochrome b (m.15452C>A), NADH dehydrogenase 1 (m.4216T>C) and NADH dehydrogenase 5 (m.13708G>A). All these gene products are involved in the oxidative phosphorylation and ATP production [8], [37]. Furthermore, a different study revealed that the m.295C>T polymorphism of haplogroup J increases the mtDNA replication *in vitro*. This polymorphism maps the mitochondrial transcription factor A binding-site but intriguingly, this mtDNA increment could not be associated with an increment in the mRNA levels of mtDNA-encoded genes [38]. We suggest that no single specific SNP is responsible for the protective effect, but it is due to a particular set of polymorphisms in haplogroup J.

There are several evidences for an association of mtDNA haplogroups with the ischemic event [25], [26], [29], but a definitive conclusion has not been reached yet. Although distribution in our control population did not differ from those in other European studies [29]–[31], an exhaustive work carried by Benn et al. in a Danish population found no differences between mitochondrial haplogroups and risk for ischemic cardiovascular disease [26]. Therefore, our Spanish population cannot be directly extrapolated to other Northern European populations. Also, Kofler et al. found a higher frequency of haplogroup T among coronary artery disease patients from Austria [30]. In our work we did not find differences in the frequency of haplogroup T. These works emphasize the difficulty of finding reproducible mitochondrial genome associations with ischemic heart disease. The differing results of these studies might arise from geographic specificity for some mtDNA SNPs and clades.

Results emerging from many studies have provided insights concerning different energy efficiency between haplogroups J and H. Haplogroup J could have lower oxygen consumption than H [11], [39], with lower efficiency in the electronic respiratory chain and low ATP and ROS production. Consequently, cells with mitochondrial haplogroup H undergo more mitochondrial oxidative damage than those carrying the J haplogroup [11]. Because the heart has the highest oxygen uptake rate in the body, we speculate that minor differences in energy efficiency might lead to major physiological effects. The present study focuses on investigating whether differences among haplogroups have consequences for ischemic cardiomyopathy development. Atherosclerosis in coronary arteries is the major cause of ischemic cardiomyopathy, and it is well established that inflammation is a key mechanism in the pathogenesis of atherosclerosis [2]. A persistent or continuously repeated insult leads to chronic inflammation that results in tissue destruction and organ dysfunction. ROS production plays a decisive role in tissue inflammation, and consequently, in atherosclerosis [40]. Endothelial cells, smooth muscle cells and macrophages are sources of ROS that oxidize low-density lipoprotein (LDL). The passage of oxidized LDL through the endothelium into the artery wall is the first step of a cascade that ultimately leads to formation of the fibrous plaque that protrudes into the arterial lumen and causes ischemia [41]. Because haplogroup H might produce more ROS than other haplogroup variants, we suggest that this precipitates the first steps in this inflammation process. In individuals with haplogroup H, the high ROS production from cells involved in the course of inflammation accelerates the catastrophic cycle of atherothrombosis. Conversely, low ROS production on cells with haplogroup J might produce the opposite effect.

It is clear that both aetiology and pathology of ischemic cardiomyopathy are complex, and the mitochondrion plays a critical role in this process. Mitochondrial haplogroups may act synergistically with other nuclear genetic factors, proteins and environmental components, which are all epistatic factors contributing to ischemic cardiomyopathy. According to the suggested criteria for establishing positive replication a similar population should be studied [42]. Although this work showed significant results, a limitation of the present study is the lack of a replication study in another related population. However, the large number of clinically well assessed patients and controls allowed us to exclude possible false positive results. A replica of our study is quite demanding, owing to difficulties in enrolling another comparable large number of patients. Nevertheless, IC patients and controls have been recruited in a relatively large geographic area thus avoiding possible bias related to founder effect or population heterogeneity.

In summary, our results in a Spanish population show suggestive evidence for the association of the mitochondrial haplogroups H and J as risk and protection factors for ischemic cardiomyopathy respectively, in a Spanish population. Future analysis of the full sequenced mtDNA in these haplogroups and their phenotypic analysis will yield additional insights towards therapeutic targets for ischemic cardiomyopathy pathogenesis.

## Supporting Information

Table S1 **Mitochondrial haplogroup frequencies (%) stratified by classical risk factors for ischemic cardiomyopathy development.**
(DOC)

Table S2 **Frequencies of control population in this study and in other European studies.**
(DOC)
