# Supplementary material for: Mitochondrial Haplogroups H and J: Risk and Protective Factors for Ischemic Cardiomyopathy
Source: PLoS One. 2012 Aug 28;7(8):e44128. doi: 10.1371/journal.pone.0044128 (PMC3429437; doi:10.1371/journal.pone.0044128)
Supplement: Table S1 — Mitochondrial haplogroup frequencies (%) stratified by classical risk factors for ischemic cardiomyopathy development. (DOC) [file pone.0044128.s001.doc]

**SUPPORTING INFORMATION**

**Table S1.** Mitochondrial haplogroup frequencies (%) stratified by classical risk factors for ischemic cardiomyopathy development.

| **Haplogroups** | **Hypercholesterolemia** | | **Hypertension** | | **Diabetes** | | **Smoking habit** | |
| --- | --- | --- | --- | --- | --- | --- | --- | --- |
|  | **C** | **IC** | **C** | **IC** | **C** | **IC** | **C** | **IC** |
| H | 46 (41.1) | 63 (43.8) | 51 (40.8) | 75 (43.9) | 29 (38.7) | 47 (45.6) | 28 (45.9) | 42 (43.8) |
| U | 19 (17.0) | 21(14.6) | 19 (15.2) | 25 (14.6) | 12 (16.0) | 15 (14.6) | 12 (19.7) | 17 (17.7) |
| J | 14 (12.5) | 15 (10.4) | 17 (13.6) | 17 (9.9) | 10 (13.3) | 11 (10.7) | 6 (9.8) | 10 (10.4) |
| T | 12 (10.7) | 16 (11.1) | 16 (12.8) | 21 (12.3) | 9 (12.0) | 11 (10.7) | 5 (8.2) | 9 (9.4) |
| K | 2 (1.8) | 4 (2.8) | 2 (1.6) | 6 (3.5) | 5 (6.7) | 5 (4.9) | 4 (6.6) | 7 (7.3) |
| W | 1 (0.9) | 4 (2.8) | 1 (0.8) | 4 (2.3) | 1 (1.3) | 1 (1.0) | 1 (1.6) | 3 (3.1) |
| V | 2 (1.8) | 5 (3.5) | 3 (2.4) | 7 (4.1) | 2 (2.7) | 2 (1.9) | 2 (3.3) | 2 (2.1) |
| I | 3 (2.7) | 3 (2.1) | 3 (2.4) | 4 (2.3) | 0 (0.0) | 3 (2.9) | 0 (0.0) | 1 (1.0) |
| X | 5 (4.5) | 6 (4.2) | 4 (3.2) | 6 (3.5) | 2 (2.7) | 3 (2.9) | 1 (1.6) | 1 (1.0) |
| HV | 3 (2.7) | 1 (0.7) | 4 (3.2) | 2 (1.2) | 4 (5.3) | 2 (1.9) | 0 (0.0) | 2 (2.1) |
| OTHERS | 5 (4.5) | 6 (4.2) | 5 (4.0) | 4 (2.3) | 1 (1.3) | 4 (3.9) | 2 (3.3) | 2 (2.1) |
| Total | 112 | 144 | 125 | 171 | 75 | 104 | 61 | 96 |

C. Controls. IC Ischemic cardiomyopathy patients. No significant differences were found for the frequencies of major cardiovascular risk factors between controls and IC patients stratified by mitochondrial haplogroups.
