# Supplementary material for: Mitochondrial Haplogroups H and J: Risk and Protective Factors for Ischemic Cardiomyopathy
Source: PLoS One. 2012 Aug 28;7(8):e44128. doi: 10.1371/journal.pone.0044128 (PMC3429437; doi:10.1371/journal.pone.0044128)
Supplement: Table S2 — Frequencies of control population in this study and in other European studies. (DOC) [file pone.0044128.s002.doc]

**SUPPORTING INFORMATION**

**Table S2.** Frequencies of control population in this study and in other European studies.

| **Haplogrups** | **This study**  **N=423** | **Finland and Sweden [29]**  **N=134** | **Austria [30]**  **N=1527** | **Italy [31]**  **N=686** |
| --- | --- | --- | --- | --- |
| H | 40.0 | 41 | 43.6 | 41 |
| U | 17.3 | 14.2 | 15.5 | 12.4 |
| J | 11.1 | 11.2 | 11.4 | 9.5 |
| T | 11.1 | 11.9 | 8.3 | 9.1 |
| K | 6.6 | 7.5 | 5.2 | 5.6 |
| W | 1.4 | 2.2 | 2.1 | 0.8 |
| V | 3.1 | 3.0 | 1.8 | 1.0 |
| I | 1.4 | 2.2 | 1.0 | 2.7 |
| X | 3.5 | 4.5 | 1.3 | 6.2 |
| SuperHV | 1.4 | … | … | … |
| Other | 3.1 | 2.2 | 9.8 | 11.3 |
